# The association between night eating syndrome and GERD symptoms among university students at An-Najah National University in Palestine: a cross-sectional study

**DOI:** 10.1186/s12876-024-03259-y

**Published:** 2024-05-17

**Authors:** Mohammad Taleb Abed, Eyad Sayyed, Obada Yamak, Qusay Abdoh, Manal Badrasawi

**Affiliations:** 1 Department of Medicine, College of Medicine and Health Sciences, An-Najah National University, Nablus, 44839 Palestine; 2 Department of Internal Medicine, GI and Endoscopy Unit, An-Najah National University Hospital, Nablus, 44839 Palestine; 3 Department of Nutrition and Food Technology, College of Medicine and Health Sciences, An- Najah National University, Nablus, 44839 Palestine

**Keywords:** Night eating syndrome (NES), Gastroesophageal reflux disease (GERD), Mediterranean diet, Lifestyle, University students

## Abstract

**Background:**

Night eating syndrome (NES) is a kind of eating disorder. NES association with gastroesophageal reflux disease (GERD) symptoms among university students is still not fully understood. We aimed to determine the relationship between NES and the presence of GERD symptoms among university students at An-Najah National University in Palestine.

**Methods:**

This study involved undergraduate students from An-Najah National University. The data were collected through online surveys from November to December 2023. The sampling frame involved voluntary sampling, as the data were collected using a structured questionnaire to collect data on sociodemographic variables, medical history, lifestyle habits, nutritional status, GERD risk, and NES. The GERD questionnaire (GerdQ) was used to assess symptoms, while the Arabic version of the validated Night Eating Questionnaire (NEQ) was used to assess night eating. Physical activity was assessed using the short form of the International Physical Activity Questionnaire (SF-IPAQ), and adherence to a Mediterranean diet was assessed using the validated Arabic version of the MEDAS. Both univariate and multivariate analyses were also conducted to assess the study hypotheses.

**Results:**

The study involved 554 participants, 59.9% female. A total of 33.4% reported GERD symptoms, with 10.3% having NES. A strong association was observed between GERD and NES and between GERD and physical activity. Night eating syndrome (AOR = 2.84, CI = 1.07–3.19), high physical activity (AOR = 0.473, CI = 1.05–3.19), and non-smoking (AOR = 0.586, CI = 1.27–7.89) were identified as independent predictors of GERD symptoms.

**Conclusion:**

This study revealed that 33.4% of undergraduate students were at risk of GERD, with night eaters having a greater risk. GERD risk was negatively associated with physical activity level and smoking status. No associations were found between GERD risk and weight status, Mediterranean diet adherence, sociodemographic factors, or sleep disturbances.

## Introduction

According to the Montreal criteria, gastroesophageal reflux disease (GERD) is a condition marked by distressing symptoms and complications resulting from regurgitation of stomach contents into the esophagus [1–4]. According to a systematic analysis of GERD epidemiology, the global pooled incidence was 13.98%, which varied substantially by region (19.55% in North America, 4.16% in China, and 22.40% in Turkey) [5]. GERD is associated with various lifestyle habits, including smoking, alcohol consumption, and mental stress. The dietary components included midnight snacking, skipping breakfast, eating rapidly, and overeating. Engaging in regular physical activity is a beneficial protective factor [6].

Night-eating syndrome (NES) is an eating disorder characterized by a delayed circadian rhythm of food consumption. It was initially observed in obese individuals who did not respond to conventional weight management methods. It is distinguished by sleeplessness or insomnia (3 times per week), morning anorexia (negligible “i.e., juice or coffee” or no consumption at regular breakfast), evening hyperphagia (eating at least 25% of daily food intake following an evening meal), and snack consumption during night awakening (> two weeks) [7]. NES is associated with several factors, including sex, obesity, depression, drug use, disruptions in leptin and melatonin levels, and increased levels of cortisol in the blood [8]. Furthermore, studies have documented connections with poor psychological and physical functioning, behaviors, and attitudes [9].

Eating disorders, such as anorexia nervosa and bulimia nervosa, can negatively impact health and lead to gastrointestinal diseases, including GERD, esophagitis, and ulcers. Night eating, which disrupts bowel movement [10] is more common in late adolescence, particularly among university students with body image issues, sleep difficulties, worry, and stress, all of which increase the likelihood of developing NES symptoms. In addition, university students are particularly susceptible to developing NES as a result of their lifestyle choices, including their excessive late-night use of technology and their consumption of caffeine, which impact their physiological balance [11]. Researches have shown that evening hyperphagia was more prevalent in young people aged 18–30 years than in the general population [12–14].

However, few studies have explored the relationship between functional gastrointestinal disorders and eating disorders [10]. Fujiwara Y. et al. (2005) found that a shorter time between dinner and bedtime was significantly linked to a higher odds ratio of GERD (*p* < 0.001), even when smoking, drinking, and body mass index were taken into account. The odds ratio (OR) for patients whose dinner-to-bedtime was less than 3 h was 7.45, compared to patients whose dinner-to-bedtime was 4 h or more. These findings were similar in individuals with nonerosive GERD and erosive esophagitis, and there was no significant difference in dinner-to-bed time intervals between the two conditions [15].

This study aimed to determine the association between GERD symptoms and night eating syndrome among university students at An-Najah National University. Despite the established link between night eating and GERD pathogenesis, this study is the first to assess the association between GERD and night eating syndrome. Furthermore, there is a lack of information in the local data regarding the incidence of GERD among university students and its related factors. This study aimed to enhance understanding of GERD and NES by exploring modifiable factors associated with these conditions. Additionally, the study examined the implementation of preventative initiatives within the healthcare system in Palestine.

## Methods

### Study design and settings

This cross-sectional study was conducted among Palestinian undergraduate students from An-Najah National University, West Bank, Palestine. The data were collected from November 2023 to December 2023.

### Sample size

The sample size was determined using a proportion for a finite population. The sample size was calculated using G Power software, considering an alpha of 0.05 (two-sided) and 80% power (Beta = 0.2). The effect size was determined using the prevalence GERD among university students from Saudi Arabia (similar study) the study found the prevalence of GERD was 23.8% [16]. The expected difference in prevalence from the aforementioned study was considered 10%. A minimum of 230 participants were required to assess the prevalence of GERD. Furthermore, the sample size calculation took into account the objective to determine the association between GERD and lifestyle and sociodemographic factors. To estimate sample size a moderate effect size of approximately Cohen’s d of 0.5, the level of significance or type I error of 0.05 (5%), and a power or type II error or (1-β) of 0.8 (80%) were set. The resultant sample size calculations all suggested that a minimum of 420 participants would be sufficient for performing the analyses. Considering the drop out due to missing data the sample size was augmented to include minimum 480 participants.

### Sampling and data collection approach

The study conducted an online survey and chose its participants through volunteer sampling, where participants are self-chosen. We collected the data by generating an online structured questionnaire on Google Forms and distributing it via the official Facebook groups of the selected university students. Furthermore, the researchers contacted university professors, soliciting their assistance in disseminating the questionnaire among their students. Participants provided informed online written consent. The questionnaire’s introduction mentioned the study objectives and emphasized the voluntary nature of participation. There were no rewards or promotions offered. The research techniques followed all applicable rules and regulations. All information was kept private and only used for research purposes. We included all university students who were undergraduates aged 18 years and older, studying at An-Najah National University in Palestine, and were willing to participate and provide all the required data. We excluded students who were previously diagnosed with GERD to reduce potential biases and confounding variables, in addition to patients with inflammatory bowel diseases (IBD), any upper gastrointestinal disease (i.e., hiatal hernia, esophagitis, or peptic ulcer), or any disease that affected nutritional status; students who were not registered during the semester of data collection; and incomplete responses or invalid data.

### Study tool

The study used the following instruments and tools to establish operational definitions for the study variables:

### GERD symptoms

The presence of GERD symptoms was assessed using a GERD questionnaire. Six items were included in the new GerdQ. In a previous study, the GerdQ reliability was 0.81 for patients and 0.90 for healthy controls; the validity was 88% [17], the sensitivity was 67%, and the specificity was 70%. Scores ranging from 0 to 3 were applied for the positive predictors, and scores ranging from 3 to 0 (reversed order, where 3 = none) for the negative predictors. The GerdQ score was calculated by adding these scores, resulting in a total score that ranged from 0 to 18 [18]. A cutoff score of 8 was used. The reliability of the GerdQ questionnaire in our sample was assessed, with a Cronbach’s alpha of 0.62.

### Night eating syndrome

A validated Arabic version of the night eating questionnaire (NEQ) was used to assess NES [19]. The NEQ is a 16-item questionnaire with a 5-point Likert scale. The cutoff criterion was 25 points. The NEQ questionnaire is highly reliable in our sample, with a Cronbach’s alpha of 0.72.

The demographic data included age, sex, academic year, discipline of study, marital status, place of residence, and economic status. Lifestyle data included smoking history (yes or no) and physical activity according to the IPAQ.

Physical activity was assessed using the validated Arabic version of the International Physical Activity Questionnaire (IPAQ-SF) [20]. The IPAQ-SF evaluates the total number of days, the duration of moderate, vigorous, or walking physical activity in the previous seven days, and the duration of sitting on weekdays. Next, we categorized the data into three categories: low, moderate, and high. The IPAQ-SF demonstrated reliability ranging from 0.71 to 0.89, indicating that the questionnaire questions were somewhat highly reliable and had a good validity [21].

Regarding medical history, participants were asked about the existence of chronic diseases. If yes, they were requested to specify the disease and medications employed. Additionally, participants were inquired about any prior surgical procedures and their frequent consumption of nutritional supplements.

Nutritional status was assessed by body mass index (BMI), self-reported height and weight, and adherence to the Mediterranean diet (MD) as a healthy diet indicator. Adherence to the MD was assessed using a validated Arabic version of the Mediterranean diet adherence scale (MEDAS), containing 14 items and dichotomous questions about adherence to MD features [22]. The MEDAS diet includes 12 questions related to the frequency of consumption of the MD food component and two questions about food intake habits related to the MD. Each item scored 1 or 0 based on participants adherence to each item. The final score ranges from 0 to 14, and a score of 9 or more indicates adequate diet adherence [23, 24].

### Statistical analysis

The Statistical Package for the Social Sciences (SPSS) was used for the statistical analysis. The data were summarized using descriptive summary measures and are expressed as the mean ± standard deviation for continuous variables and percentages for categorical variables. Any possible relationship between the dependent and independent variables was explored using an appropriate statistical significance test (binary logistic regression, t-test). A significance level of < 0.05 was used in this study. Further analysis was performed using a binary logistic regression test, and the logistic regression assumptions were checked before conducting the analysis. The multicollinearity was checked using collinearity diagnostic tests. The Hosmer-Lemeshow goodness of fit test was employed to assess how well the model fits the data.

## Results

### a. Subject recruitment

A total of 616 responses were received, 554 were included in the final analysis, and 62 participants were excluded due to duplicate responses, missing or invalid data, a diagnosis of GERD, or because the participants were postgraduate students.

### b. Demographic variables of the participants

The final analysis included a total of 554 subjects: 222 (40.1%) men and 332 (59.9%) women. Table 1 shows the sociodemographic characteristics of the participants are presented in Table 1. The majority of the participants were in the faculty of medicine and health science (37.9%), in the second academic year (22%), single (96.4%), lived in towns or villages (49.3%) and their family income ranged between 2001 and 4000 NIS (36.8%). The mean age was 20.7 ± (2.4) years.

### c. Medical history and lifestyle characteristics

As shown in Table 1, the percentage of smokers was 18.2%. Most of the participants had no chronic diseases (95.7%), had never undergone surgery (75.1%), and hadn’t used drugs continuously (94.2%). The most commonly reported comorbidity was asthma; most of the medications used were supplements. Regarding physical activity, 246 (44.4%) of the participants were inactive, 136 (24.5%) were moderately active, and 172 (31%) were very active.

### d. Nutrition status of the participants

As shown in Table 1, the results revealed that the majority of the participants (332; 59.9%) had a normal weight, 47 (8.5%) were underweight, 126 (22.7%) were overweight, and 49 (8.8%) were obese. More than 65% of the participants had moderate adherence to the MD, 9.2% had low adherence, and 142 (25.6%) had high adherence to the MD.

**Table 1:** Study participants characteristics

Variable (unit)		*n* (%)
**Sociodemographic characteristics**		
Sex	Male	222 (40.1%)
	Female	332 (59.9%)
Academic year	First	78 (14.1%)
	Second	122 (22%)
	Third	96 (17.3%)
	Fourth	120 (21.7%)
	Fifth	74 (13.4%)
	Sixth	64 (11.6%)
Faculty	Faculty of medicine and health science	210 (37.9%)
	Faculty of Theoretical and Applied Science	180 (32.5%)
	Faculty of Economic Sciences	164 (29.6%)
Marital status	Single	534 (96.4%)
	Married	20 (3.6%)
Mean income NIS^*^/month	Less than 2000	44 (7.9%)
	2001–4000	204 (36.8%)
	4001–6000	172 (31%)
	Above 6000	134 (24.2%)
Place of living	City	250 (45.1%)
	Town\village	273 (49.3%)
	Refugee camp	31 (5.6%)
**Medical history and lifestyle**		
Smoker	Yes	101 (18.2%)
	No	453 (81.8%)
Chronic disease	Yes	24 (4.3%)
	No	530 (95.7%)
Surgery	Yes	138 (24.9%)
	No	416 (75.1%)
Medications	Yes	32 (5.8%)
	No	522 (94.2%)
Physical Activity (IPAQ)	Low	246 (44.4%)
	Moderate	136 (24.5%)
	High	172 (31%)
**Nutritional status of participants**		
BMI	Underweight (below 18.5)	47 (8.5%)
	Normal (18.5–24.9)	332 (59.9%)
	Overweight (25-29.9)	126 (22.7%)
	Obese (30 or above)	49 (8.8%)
The Adherence to Mediterranean diet MEDAS	Low	51 (9.2%)
	Moderate	361 (65.2%)
	High	142 (25.6%)

*1NIS = 0.27 United States Dollar

*Abbreviations*: NIS: New Israeli Shekel; IPAQ: International Physical Activity Questionnaire; BMI: body mass index; MEADS: Mediterranean diet adherence scale

### Frequency of GERD symptoms

**Fig. 1:**
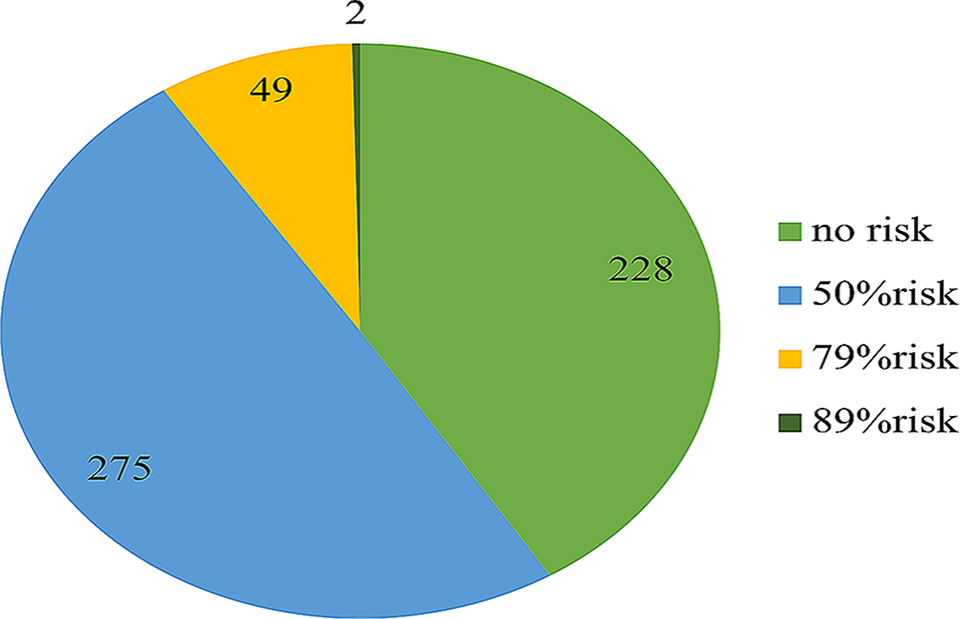
Participants distribution according to GERD risk

As shown in (Fig. 1), two hundred twenty-eight participants (41.2%) were not at risk of having GERD, and 275 participants (49.6%) had a 50% likelihood of having GERD, which was the highest value in our study. Forty-nine participants (8.8%) had a 79% likelihood of having GERD, and only 2 participants (0.4%) had an 89% likelihood of having GERD. The participants were further classified into two groups based on their risk of GERD. Using a cutoff score of 8, it was found that 369 participants (66.6%) did not have GERD, while 185 participants (33.4%) had GERD.

### e. Frequency of NES

**Fig. 2:**
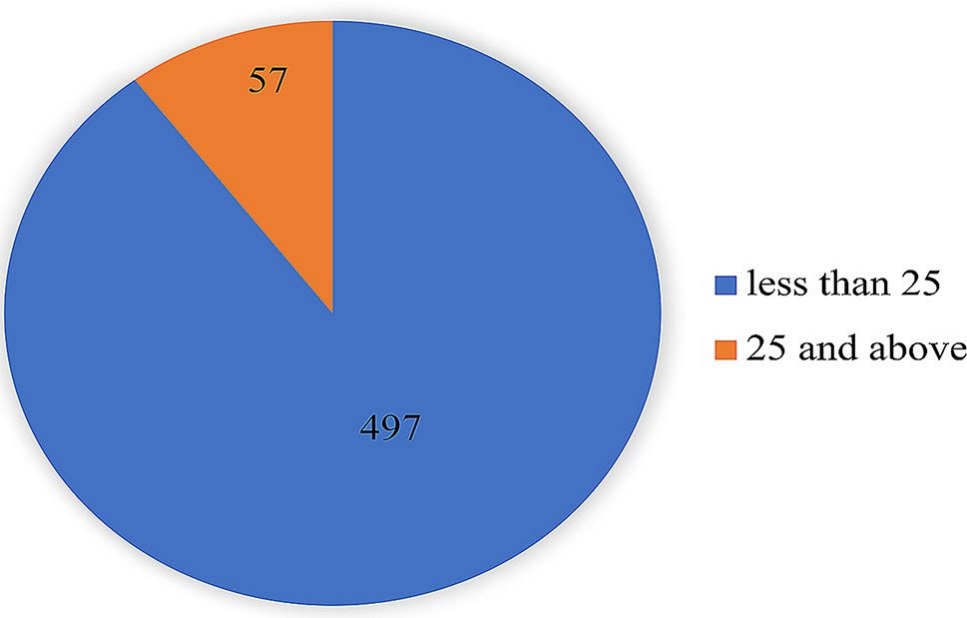
Prevalence of night eating syndrome

Regarding the NES, the results demonstrated that 57 participants (10.3%) had a score of 25 or above on the NEQ, and 497 (89.7%) participants were normal eaters, as shown in (Fig. 2).

### f. Associations of GERD with the study variables

As shown in Table 2, the results indicated that none of the sociodemographic characteristics was significantly associated with GERD according to the Chi-square test. In regard to GERD association with the NES, the findings indicate that individuals who were night eaters have a significantly greater chance of developing GERD (64.9%) than do those who weren’t (29.8%). These results demonstrated a clear and robust relationship between the NES score and GERD risk, with a statistically significant p-value of less than 0.001. The association of GERD with lifestyle and dietary factors revealed that Among the lifestyle factors, physical activity was found to be associated with a reduced risk of GERD. Participants who engaged in high levels of physical activity had a lower risk of developing GERD (26.7%) than those who were sedentary (32.5%) and moderately physically active participants (43.4%), *P* < 0.001, according to the Chi-square test. None of the other lifestyle or nutritional factors, such as body mass index, adherence to the MD, or smoking, were significantly associated with GERD.

**Table 2:** Association of GERD with sociodemographic factors

Categories		GERD *N* (%)		OR	CI	*P* value
		No risk	Have risk			
Sex	Male (ref)	227 (68.4%)	105(31.6%)	0.82	0.851-1.74	0.281
	Female	142 (64.0%)	80 (36.0%)			
Place of living	City (ref)	156 (62.4%)	94 (37.6%)	0.83	0.391–1.78	0.053
	Town\village	195 (71.4%)	78 (28.6%)			
	Refugee camp	18 (58.1%)	13 (41.9%)			
Mean income (NIS/month)	Less than 2000 (ref)	27 (61.4%)	17 (38.6%)	1.09	0.54–2.25	0.573
	2001–4000	142 (69.6%)	62 (30.4%)			
	4001–6000	115 (66.9%)	57 (33.1%)			
	More than 6000	85 (63.4%)	49 (36.6%)			
Academic year	First	53 (67.9%)	25 (32.1%)	1.04	0.51–2.11	0.265
	Second	88 (72.1%)	34 (27.9%)			
	Third	67 (69.8%)	29 (30.2%)			
	Forth	75 (62.5%)	45 (37.5%)			
	Fifth	42 (56.8%)	32 (43.2%)			
	Sixth	44 (68.8%)	20 (31.3%)			
Faculty	Faculty of medicine and health science (ref)	133 (63.3%)	77 (36.7%)	0.758	0.49–1.17	0.471
	Faculty of Theoretical and Applied Science	122 (58%)	58 (32.2%)			
	Faculty of Economic Sciences	114 (69.5%)	50 (30.5%)			
Marital status	Single (ref)	355 (66.5%)	179 (33.5%)	1.42	0.565–3.71	0.450
	Married	14 (70.0%)	6 (30.0%)			
NES	Normal eaters (ref)	349 (70.2%)	148 (29.8%)	4.36	2.43–7.72	0.000*
	Night eaters	20 (35.1%)	37 (64.9%)			
BMI	Underweight (ref)	32 (68.1%)	15 (31.9%)	0.882	0.377-2.1	0.481
	Normal	225 (67.8%)	107 (32.2%)			
	Overweight	80 (63.5%)	46 (36.5%)			
	Obese	32 (65.3%)	17 (34.7%)			
Adherence to Mediterranean diet	Low (ref)	29 (56.9%)	22 (43.1%)	0.941	0.68–1.86	0.704
	Moderate	248 (68.7%)	113 (21.3%)			
	High	92 (64.8%)	50 (35.2%)			
Physical Activity	Low (ref)	166 (67.5%)	80 (32.5%)	2.1	0.85–2.03	0.008*
	Moderate	77 (56.6%)	59 (43.4%)			
	High	126 (73.3%)	46 (26.7%)			
Smoking	No (ref)	308 (68.0%)	145 (32.0%)	1.39	0.89–2.17	0.144
	Yes	61 (60.4%)	40 (39.6%)			

Differences were considered not significant at *p* > 0.05 according to the binary logistic regression * Significant at *P* < 0.01 according to the binary logistic regression *Abbreviations*: NES: night eating syndrome; GERD: Gastroesophageal reflux disease. BMI: Body mass index

The binary logistic models included all the significant predictors found in the univariate analysis (NES and physical activity) and the factors theoretically associated with GERD risk but did not show a significant association in this study: BMI and smoking. The results showed that this model fulfilled the assumptions of the analysis (the multicollinearity was violated, as indicated by correlation coefficients < 0.7 for all of the variables in the model). The Hosmer and Lemeshow test revealed that the model’s goodness of fit was acceptable (*p* = 0.221), the Cox and Snell R-square was 0.147, and the Nagelkerke R-square was 0.196. According to this model, NES, physical activity, and smoking were significant predictors of GERD risk (*p* < 0.05), i.e., being a night eater increases the risk of developing GERD (AOR = 2.84, *p* < 0.001, CI = 1.07–3.19); being highly physically active and nonsmoking lowers the risk of developing GERD (AOR = 0.743, *p* < 0.001, CI = 1.05–3.19); (AOR = 0.586, *p* < 0.05, CI = 1.27–7.89), respectively, as shown in Table 3.

**Table 3:** GERD predictors

Factors	*P*-value	AOR	95% CI (lower-upper)	*P*-value
Night eater (reference: normal)	0.000*	2.84	(1.07–3.19)	0.000*
High Physical Activity (IPAQ) (reference: moderate and low)	0.000*	0.743	(1.05–3.19)	
BMI (continuous)	0.227	0.982	(1.22–5.84)	
Being nonsmoker (reference: smoker)	0.013*	0.586	(1.27–7.89)	

* *p* < 0.05 according to binary logistic regression

*Abbreviation*: AOR: adjusted odds ratio. CI: confidence interval

## Discussion

This is the first study to determine the association between GERD risk and NES among Palestinian university students. Therefore, this study provides essential information for proposing other interventional and educational programs in this field.

The results of this study revealed that one-third of university students at An-Najah National University had GERD; these findings were consistent with other findings at the Ethiopian (32.1%) [4] and the Middle Eastern universities (up to 33.1%) [25] but are higher than those of previous studies at the Saudi Arabian (21.2-29.3%) [26, 27] and the Egyptian universities (17.1%) [28]. Many factors, such as sample size, age, sex, evaluation tools, GERD definition and diagnosis, will affect the incidence of GERD among participants. Nevertheless, there were a differences in the results between the current study and the other studies. For example, there were differences in the tools and the population, which may be correlated to the permanent high-stress level Palestinians witness.

The current study showed that 10.3% of university students at An-Najah National University exhibited symptoms of NES. Previous research at Palestinian universities indicated a higher prevalence of NES (29.7%) among students. Furthermore, it was observed that the occurrence of NES was linked to mental issues, such as physiological distress, emotional disfunction, and cognitive disorder [7]. This variation can be attributed to that the previous research has been conducted during the pandemic of COVID-19, wherein the majority of individuals had a disrupted lifestyle. Furthermore, Palestinian university students have shown a high level of physiological distress, such as anxiety and depression, of up to 72.1%, according to Radwan et al. [29]. Additional research is required to have a more comprehensive knowledge of the pathways that connect physiological discomfort and NES in Palestinian youth. However, our finding is consistent with other findings at Turkish universities (9.5%) [30] but is higher than that of previous studies in the USA (4.2%) [16] and slightly lower than that of an earlier Brazilian study (16.8%) [31]. These results show variability in NES prevalence at universities worldwide, partly due to differences in sample size and participants’ variant habits that were affected by their cultures.

To the best of our knowledge, this study is the first to determine the association between GERD and NES worldwide. Our findings revealed that night eater participants had a nearly threefold higher tendency to develop GERD compared to day eater participants. In contrast, being highly physically active reduces the risk of having GERD by a factor of one, while not smoking decreases the risk by almost a half, which makes smoking a predictor of GERD. It’s worth noting the discrepancy observed in the protective interpretation of physical activity, BMI, and non-smoking (AOR < 1) and their confidence intervals indicating risk (> 1), which may be attributed to several factors, including sample size, confounding variables, measurement error, model specification, or interactions between variables. These inconsistencies point out the complex relationship between these variables and their outcomes, requiring perhaps more investigation to get an answer.

Many studies suggest a correlation between eating disorders and GERD symptoms such as anorexia nervosa [32] and bulimia nervosa [33], which suggests that self-induced vomiting, binge eating, and GERD are not significantly correlated [34]. Many others propose that late-night eating could exacerbate GERD symptoms [35, 36] as evidenced by an increase in gastric acid production [37]. Our results suggest a significant correlation between GERD symptoms and night eating syndrome, which was consistent with the fact that NES is an eating disorder that exacerbates late-night eating.

Our study revealed no significant correlation between sex and GERD incidence. This finding was consistent with those of previous studies [38, 39]. However, some recent studies have shown that there was an association [40, 41]. We did not analyze the age association with GERD in our study because of the narrow spectrum of participants’ ages; however, there were differences in the results worldwide; for example, some articles have suggested that there was an association between age and GERD [42, 43] while others haven’t found such an association [44].

In our study, participants’ BMI did not correlate with GERD incidence compared to that of another study, which showed that the incidence of GERD was greater among overweight people [45]. Moreover, a study conducted in India revealed that GERD symptoms increased in frequency, severity, and prevalence as BMI increased [46]. This difference could be mainly due to the longer duration during which obesity affects a person’s body to develop GERD symptoms by increasing abdominal pressure, which relaxes the lower esophageal sphincter, thus exposing the esophageal mucosa to gastric content and causing GERD symptoms. Most of our obese participants are young and not yet affected by their obesity. In addition, most of our participants’ BMIs were normal or underweight, possibly because of the sample accuracy, self-reported weight measurements, and a low range of participant weight variations.

Only physical activity (IPAQ) was significantly associated with lifestyle variables (smoking, dietary pattern, and physical activity), as shown above. Surprisingly, univariate analysis revealed no correlation between GERD and smoking; however, multiple studies have shown that smoking and GERD are associated [47, 48] but another study has shown that these associations are controversial [49] which is consistent with our study. This may be because of the unbalanced sample size between smokers and nonsmokers.

Our study assessed the Mediterranean diet as a lifestyle factor, not a meal pattern or content. Furthermore, we analyzed the relationship between GERD incidence and physical activity level and found a significant correlation between both; this finding was consistent with the findings of other studies [50, 51]. Conversely, a Polish study found no association between the level of daily physical activity and GERD [52]. The positive association between physical activity and GERD in our findings suggests that engaging in physical activity might have the potential to reduce the risk of developing GERD or reducing its symptoms. It also highlights the importance of promoting physical activity as a part of GERD prevention and management.

### Strength and limitations

The study’s strengths included the use of a large sample of respondents, a thorough questionnaire covering a variety of variables, and widely used, validated, and reliable instruments. However, this study has several limitations. The utilization of a cross-sectional methodology poses difficulties in precisely establishing the causal link between GERD and the NES. The data were obtained via self-report questionnaires, which may be subject to recall bias and might influence the findings.

## Conclusion

Gastroesophageal reflux disease risk was strongly prevalent among Palestinian undergraduate students; where 33.4% of participants in the present study were identified as being at risk of GERD. GERD risk was significantly greater among night eaters than among day eaters. Among the other associated factors, GERD was not associated with any of the sociodemographic factors.

The risk of GERD was notably reduced among participants who engaged in high levels of physical activity; however, smoking was not associated with GERD. Regarding nutritional factors, no association was found between GERD risk and weight status, as shown by BMI categories or dietary patterns, as indicated by adherence to the Mediterranean diet (MD).

## Data Availability

The dataset supporting the conclusions of this article is available upon request from the corresponding author.

## Abbreviations

BMI: Body Mass Index
COVID-19: Corona virus disease
GERD: Gastroesophageal Reflux Disease
IBD: Inflammatory Bowel Diseases
IPAQD: International Physical Activity Questionnaire
IPAQ-SF: International Physical Activity Questionnaire Short form
MD: Mediterranean Diet;
MEDAS: Mediterranean Diet Adherence Scale
NEQ: Night Eating Questionnaire
NES: Night Eating Syndrome
